# Association between systemic immune-inflammatory index and diabetes mellitus: mediation analysis involving obesity indicators in the NHANES

**DOI:** 10.3389/fpubh.2023.1331159

**Published:** 2024-01-10

**Authors:** Yongze Chen, Ruixian Huang, Zhenhua Mai, Hao Chen, Jingjing Zhang, Le Zhao, Zihua Yang, Haibing Yu, Danli Kong, Yuanlin Ding

**Affiliations:** ^1^Department of Epidemiology and Medical Statistics, School of Public Health, Guangdong Medical University, Dongguan, China; ^2^Department of Gastroenterology, Affiliated Hospital of Guangdong Medical University, Zhanjiang, China; ^3^Department of Critical Care Medicine, Affiliated Hospital of Guangdong Medical University, Zhanjiang, China

**Keywords:** systemic immune-inflammatory index, obesity indicator, diabetes mellitus, health and nutrition examination survey, mediation analysis

## Abstract

**Background:**

Inflammation and obesity have been widely recognized to play a key role in Diabetes mellitus (DM), and there exists a complex interplay between them. We aimed to clarify the relationship between inflammation and DM, as well as the mediating role of obesity in the relationship.

**Methods:**

Based on the National Health and Nutrition Examination Survey (NHANES) 2005–2018. Univariate analyses of continuous and categorical variables were performed using *t*-test, linear regression, and *χ*2 test, respectively. Logistic regression was used to analyze the relationship between Systemic Immune-Inflammatory Index (SII) or natural logarithm (Ln)-SII and DM in three different models. Mediation analysis was used to determine whether four obesity indicators, including body mass index (BMI), waist circumference (WC), visceral adiposity index (VAI) and lipid accumulation product index (LAP), mediated the relationship between SII and DM.

**Results:**

A total of 9,301 participants were included, and the levels of SII and obesity indicators (BMI, WC, LAP, and VAI) were higher in individuals with DM (*p* < 0.001). In all three models, SII and Ln-SII demonstrated a positive correlation with the risk of DM and a significant dose–response relationship was found (*p*-trend <0.05). Furthermore, BMI and WC were associated with SII and the risk of DM in all three models (*p* < 0.001). Mediation analysis showed that BMI and WC mediated the relationship between SII with DM, as well as Ln-SII and DM, with respective mediation proportions of 9.34% and 12.14% for SII and 10.23% and 13.67% for Ln-SII (*p* < 0.001).

**Conclusion:**

Our findings suggest that increased SII levels were associated with a higher risk of DM, and BMI and WC played a critical mediating role in the relationship between SII and DM.

## 1 Introduction

Diabetes mellitus (DM) has emerged as a global pandemic, affecting hundreds of millions of individuals worldwide, with its sustained increase constituting one of the major public health challenges (1). Total diabetes prevalence primarily reflects type 2 diabetes mellitus (T2DM), which in 2021 accounted for 96.0% of diabetes cases worldwide (1). Those with DM often confront various chronic complications, including cardiovascular diseases, stroke, visual impairments, neuropathy, kidney dysfunction, and more, which may result in severe disability and premature mortality (1, 2). Overall, the management of DM remains suboptimal.

While the precise etiology and mechanisms of DM remain incompletely elucidated, it is unequivocal that inflammation plays a pivotal role in the occurrence and development of DM (3, 4). Research has demonstrated that pro-inflammatory cytokines, including tumor necrosis factor-alpha (TNF-α) and interleukin-6 (IL-6), induce insulin resistance in target tissues such as muscle and liver by disrupting insulin signaling pathways, consequently impairing glucose uptake and utilization, ultimately elevating blood glucose levels (5, 6). Furthermore, inflammatory cytokines can induce beta cell stress and apoptosis, leading to decreased insulin production and secretion (6). The Systemic Immune-Inflammatory Index (SII) is a crucial indicator that reflects the systemic inflammatory status by combining various components of peripheral blood, offering a comprehensive assessment of systemic inflammation (7). Elevated SII levels are indicative of a pro-inflammatory state, which associated with a higher risk of several chronic disease (8, 9). Therefore, understanding the significance of SII in DM research is pivotal, as it provides a comprehensive view of inflammatory state and its potential role in the development and progression of the disease.

On the other hand, obesity is closely linked to DM, particularly T2DM, and is recognized as a leading risk factor for the condition (10). In obese individuals, the enlargement of fat cells leads to increased glucose consumption, subsequently inhibiting insulin action and causing insulin resistance, potentially resulting in elevated blood glucose levels (11). Moreover, obese individuals are more susceptible to metabolic abnormality, further increasing the risk of T2DM (12). In addition to conventional measures such as body mass index (BMI) and waist circumference (WC), two novel obesity indicators, namely the visceral adiposity index (VAI) and the lipid accumulation product index (LAP), are also low-cost indicators and used to reflect obesity from different perspectives (13, 14). Among them, BMI is widely used to evaluate “total obesity” and WC can provide insights into abdominal or central obesity (15). VAI aims to accurately reflect the distribution of fat in the body, while LAP emphasizes the lipid accumulation associated with central obesity (13). The comprehensive use of these obesity indicators provides a wealth of data for in-depth analysis and conclusions in research.

The relationship between inflammation and obesity is a central focus of DM research, characterized by their intricate interplay, especially for T2DM. Research suggests that obesity serves as a key driver of chronic low-grade inflammation, with adipose tissue in obese individuals releasing a spectrum of pro-inflammatory cytokines, including TNF-α, IL-6, and others (16). These factors exacerbate systemic inflammation, disrupting the insulin signaling pathway, reducing the responsiveness of the tissues to insulin, and ultimately leading to elevated blood glucose levels (16). Additionally, under this inflammatory condition, appetite and metabolic regulation may be disturbed, potentially resulting in increased food intake, thereby exacerbating the obesity and further contributing to the development of DM (17, 18).

In summary, inflammation and obesity are intricately linked to DM. To further explore the relationship between inflammatory status, obesity, and DM, we evaluated the association between inflammation status and DM in adult population based on the National Health and Nutrition Examination Survey (NHANES), and conducted mediation analyses to examine the possible mediating role of multiple obesity indicators in the link between inflammation status and DM.

## 2 Methods

### 2.1 Participants and study design

**Figure 1:**
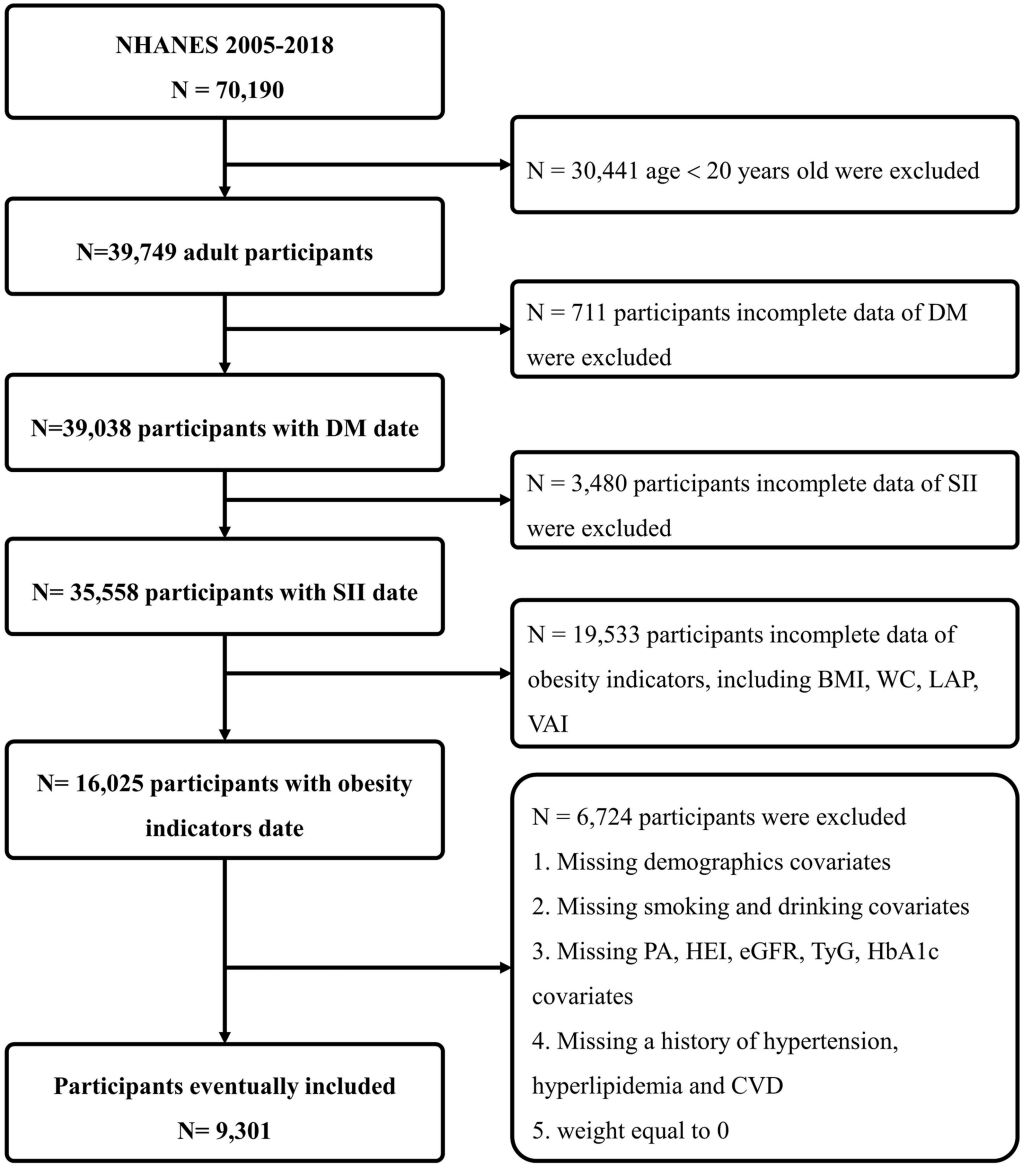
Flowchart of participants selection.

NHANES is a comprehensive cross-sectional study initiated and executed by the National Center for Health Statistics (NCHS), with the primary aim of gathering data on the health and nutritional status of both adults and children in the United States. NHANES adopts a sophisticated, stratified, multi-stage probability survey design, annually recruiting a demographically representative sample of the U.S. populace, with all participants providing informed consent. For our analysis, we utilized NHANES data from seven survey cycles conducted between 2005 and 2018, resulting in a substantial sample size of 70,190 participants. Exclusions were made based on the following criteria: individuals aged <20 years (*n* = 30,441); those with missing DM health questionnaire data (*n* = 711); those with missing SII data (*n* = 3,480); those with incomplete data for obesity indicators, including BMI, WC, LAP, and VAI (*n* = 19,553); participants without demographics [gender, race, education level, marital status and poverty index ratio (PIR)], or without information on smoking, alcohol use, specifics on physical activities (PA), healthy eating index (HEI), laboratory tests data [estimated glomerular filtration rate (eGFR), triglyceride and glucose index (TyG) and glycosylated hemoglobin (HbA1c)], or medical history data [hypertension, hyperlipidemia and cardiovascular diseases (CVD)], or those with a weight of 0 (*n* = 6,724). Finally, we compiled a definitive dataset consisting of 9,301 participants, representing a weighted number of 211,667,721 participants, for our comprehensive statistical analysis (Figure 1).

### 2.2 Exposure variable

SII is determined from complete blood cell count results, and the laboratory methodology for the complete blood count test is detailed on the NHANES website (19). The standard formula for calculating SII is presented as SII = P × N/L, wherein P, N, and L correspond to the counts of platelets, neutrophils, and lymphocytes in peripheral blood, respectively (20). Within the scope of our investigation, SII is identified as the exposure variable.

### 2.3 Assessment of mediators

Following standardized procedures, skilled healthcare professionals at the mobile examination center employed rigorous techniques and state-of-the-art equipment to measure the body height (BH), body weight (BW), and WC of all recruited participants. During the assessment of height and weight, participants were outfitted in minimal attire, including underwear, disposable paper robes, and foam slippers. Waist circumference was precisely gauged using a tape measure at the narrowest point between the lowest rib and the iliac crest. After a mandatory fasting period of at least 8 h, blood samples were systematically collected from participants for a comprehensive examination, encompassing triglyceride (TG), high-density lipoprotein cholesterol (HDL-C). The NHANES website provides detailed information about the examination process (19). The formulas for calculating BMI, LAP, and VAI are outlined below (21):

BMI = BW (kg)/BH^2^ (m)

LAP = (WC [cm] – 65) × TG [mmol/L] in males

LAP = (WC [cm] – 58) × TG [mmol/L] in females

VAI = [WC [cm] / 39.68 + (1.88 × BMI [kg/m^2^])] × (TG [mmol/L]/ (1.03 × 1.31 / HDL [mmol/L])) in males

VAI = [WC [cm] / 36.58 + (1.89 × BMI [kg/m^2^])] × (TG [mmol/L]/ (0.81 × 1.52 / HDL [mmol/L])) in females

### 2.4 Outcome variable

The DM diagnostic criteria in this study include: (1) Physician-diagnosed diabetes; (2) HbA1c > 6.5%; (3) Fasting blood glucose ≥7.0 mmoL/L; (4) Random blood glucose ≥11.1 mmoL/L; (5) Two-hour Oral Glucose Tolerance Test (OGTT) blood glucose ≥11.1 mmoL/L; (6) Use of diabetes medication or insulin.

### 2.5 Study covariates

In line with previous studies, our multivariable models take into account potential confounding variables associated with the correlation between SII and DM. These variables encompass age (years), gender (female or male), race (non-Hispanic black, Hispanic white, Mexican American, other Hispanic, other race), education level (less than high school, completed high school, and more than high school), marital status (never married, partner or married, separated and divorced, widowed), income status based on PIR (< 1, 1–4, > 4), PA (< 600 MET-min/week, 600–3,000 MET-min/week, ≥ 3,000 MET-min/week), smoking status (never, former smoker, current smoker), alcohol use (never, former drinker, current drinker), hypertension (no or yes), hyperlipidemia (no or yes), CVD (no or yes), HEI, eGFR (mL/min), TyG and HbA1c (%).

### 2.6 Statistical analysis

Weighted analysis of NHANES data was performed using the “survey” package of R 4.2.3. Baseline characteristics of the population were presented as mean ± standard deviation for continuous variables, while categorical variables were expressed frequency (percentage). Differences in characteristics between the DM and non-DM groups were evaluated using Student’s *t*-test (for continuous variables) or chi-square test (for categorical variables). The linear regression (for continuous variables) or chi-square test (for categorical variables) was employed to explore between-group differences in SII quartile and tertile distribution.

In fitting statistical models through three distinct approaches, weighted logistic regression was utilized to estimate the odds ratio (OR) and 95% confidence interval (CI) for the association between DM and SII quartiles. We reported the risk estimation of DM occurrence for each 1-SD (z-score) increase in SII. Three models were proposed: Model 1, which did not adjust for any confounding factors; Model 2, which adjusted for age, gender, race, education, marital status, and PIR; Model 3, which adjusted for age, gender, race, education level, marital status, PIR, PA, smoking status, drinking status, hypertension, hyperlipidemia, CVD, HEI, eGFR, TyG and HbA1c. Additionally, to assess the presence of a dose–response relationship between SII and DM across the three models, we positioned four nodes at the 5th, 35th, 65th, and 95th percentiles of the natural log-transformed SII distribution to construct restricted cubic spline models. It is worth noting that in the statistical analysis, we observed that the SII data was unevenly distributed and had obvious skew, so a natural logarithmic transformation (natural logarithm (Ln)-SII) was applied to make it more suitable for our statistical analysis.

We evaluated the correlations between four obesity indicators and used weighted multivariable regression analysis to examine the relationship between obesity indicators and SII as well as DM. Additionally, we applied mediation models based on bootstrapping calculations to investigate the direct impact of SII on DM risk and the indirect effect mediated by obesity indicators. Lastly, to address potential variations in SII categorizations, sensitivity analyses were conducted by reclassifying SII into tertiles. All statistical analyses were performed using R 4.2.3 (R Foundation for Statistical Computing, Vienna, Austria), and *p*-values <0.05 (two-tailed) were considered statistically significant.

## 3 Results

### 3.1 Characteristics of the study population

This study enrolled 9,301 participants, among whom 1,619 were diagnosed with DM (17.41%). The eligible participants with an average age of 45.63 ± 16.18 years, comprised 4,352 females and 4,949 males. In comparison to the non-DM group, the DM group exhibited higher proportions of males, non-Hispanic Black, education level less than high school, widowed, moderate-income level (PIR 1–4), low-PA level (< 600 MET-min/week), former smokers, former drinkers, and those with underlying conditions (hypertension, hyperlipidemia, and CVD) (*p* < 0.05). Additionally, individuals with DM showed higher levels of age, TyG, HbA1c, obesity indicators (BMI, WC, LAP, and VAI), along with elevated SII (*p* < 0.001). Conversely, eGFR levels were lower in the DM group (*p* < 0.001). The general characteristics of the study population are presented in Table 1.

**Table 1:** Characteristics of the study population.

Variable	Total (*n* = 9,301)	DM		χ2/t	*p* value
		No (*n* = 7,682)	Yes (*n* = 1,619)		
Age	45.63 ± 16.18	43.93 ± 15.76	57.23 ± 14.13	25.77	<0.001
Gender				0.57	0.045
Female	4,352 (0.48)	3,650 (0.48)	706 (0.46)		
Male	4,949 (0.52)	4,032 (0.52)	917 (0.54)		
Race				10.75	<0.001
Non-Hispanic Black	1,762 (0.10)	1,390 (0.09)	372 (0.13)		
Non-Hispanic White	4,367 (0.71)	3,725 (0.72)	642 (0.66)		
Mexican American	1,361 (0.07)	1,073 (0.07)	288 (0.09)		
Other Hispanic and other	1,811 (0.12)	1,494 (0.12)	317 (0.12)		
Education level				16.46	<0.001
Less than high school	1,802 (0.13)	1,381 (0.12)	421 (0.17)		
Completed high school	2,105 (0.22)	1,699 (0.22)	406 (0.27)		
More than high school	5,394 (0.65)	4,602 (0.66)	792 (0.56)		
Married				38.26	<0.001
Never married	1,776 (0.19)	1,623 (0.20)	153 (0.09)		
Partner or married	5,746 (0.65)	4,716 (0.65)	1,030 (0.67)		
Separated or divorced	1,259 (0.12)	990 (0.11)	269 (0.15)		
Widowed	520 (0.04)	353 (0.03)	167 (0.08)		
PIR				7.93	0.001
< 1	1,728 (0.12)	1,418 (0.12)	310 (0.12)		
1–4	4,853 (0.48)	3,941 (0.47)	912 (0.54)		
> 4	2,720 (0.40)	2,323 (0.40)	397 (0.34)		
PA				17.61	<0.001
< 600	2,172 (0.23)	1,722 (0.22)	450 (0.28)		
600–3,000	3,669 (0.40)	2,982 (0.40)	687 (0.44)		
> 3,000	3,460 (0.37)	2,978 (0.38)	482 (0.28)		
Smoking status				18.15	<0.001
Never	5,095 (0.55)	4,287 (0.55)	808 (0.50)		
Former smoker	2,324 (0.25)	1,783 (0.24)	541 (0.34)		
Current smoker	1,882 (0.20)	1,612 (0.20)	270 (0.16)		
Alcohol use				40.46	<0.001
Never	1,050 (0.09)	818 (0.08)	232 (0.13)		
Former drinker	1,335 (0.12)	976 (0.11)	359 (0.19)		
Current drinker	6,916 (0.79)	5,888 (0.81)	1,028 (0.69)		
Hypertension				375.82	<0.001
No	5,699 (0.66)	5,183 (0.70)	516 (0.33)		
Yes	3,602 (0.34)	2,499 (0.30)	1,103 (0.67)		
Hyperlipidemia				202.03	<0.001
No	2,761 (0.31)	2,555 (0.34)	206 (0.11)		
Yes	6,540 (0.69)	5,127 (0.66)	1,413 (0.89)		
CVD				189.56	<0.001
No	8,481 (0.93)	7,187 (0.95)	1,294 (0.81)		
Yes	820 (0.07)	495 (0.05)	325 (0.19)		
HEI	50.57 ± 13.76	50.55 ± 13.87	50.72 ± 13.01	0.35	0.729
eGFR	96.36 ± 20.42	97.67 ± 19.59	87.40 ± 23.46	−13.53	<0.001
TyG	8.56 ± 0.66	8.47 ± 0.60	9.16 ± 0.74	24.95	<0.001
HbA1c	5.54 ± 0.87	5.34 ± 0.36	6.93 ± 1.67	29.85	<0.001
BMI	28.63 ± 6.56	28.05 ± 6.24	32.60 ± 7.24	16.62	<0.001
WC	98.30 ± 21.10	96.56 ± 15.59	110.22 ± 16.06	22.76	<0.001
LAP	54.80 ± 55.98	49.31 ± 48.84	92.29 ± 81.48	14.64	<0.001
VAI	1.91 ± 2.20	1.76 ± 1.87	2.99 ± 3.54	9.93	<0.001
SII	515.05 ± 295.89	506.07 ± 289.08	576.36 ± 323.43	6.22	<0.001
Quartiles of SII				13.60	<0.001
Q1	2,331 (0.23)	1,980 (0.24)	351 (0.17)		
Q2	2,320 (0.25)	1,934 (0.26)	386 (0.22)		
Q3	2,325 (0.26)	1,927 (0.26)	398 (0.27)		
Q4	2,325 (0.26)	1,841 (0.25)	484 (0.33)		
Ln-SII	6.11 ± 0.51	6.10 ± 0.51	6.22 ± 0.54	6.54	<0.001

Percentage and mean ± standard deviation were weighted. DM, diabetes mellitus; PIR, poverty index ratio; PA, physical activity; CVD, cardiovascular disease; HEI, healthy eating index; eGFR, estimated glomerular filtration rate; TyG, triglyceride and glucose index; HbA1c, glycohemoglobin; BMI, body mass index; WC, waist circumference; LAP, lipid accumulation product index; VAI, visceral adiposity index; SII, systemic immune-inflammation index; Ln, natural logarithm.

### 3.2 Characteristics of the participants according to the quartiles of SII

The characteristics of participants categorized by SII quartiles are presented in Table 2. With higher SII scores, there were increased levels of age, TyG, HbA1c, and obesity indicators (BMI, WC, LAP and VAI) (*p* < 0.05), while HEI and eGFR showed a gradual decrease (*p* < 0.05). When compared to the first quartile, participants in the second to fourth quartiles of the SII group had a higher proportion of females, non-Hispanic white, widowed, low-PA level (< 600 MET-min/week), hyperlipidemia and DM (*p* < 0.001). Furthermore, compared to the first quartile, participants in the third and fourth quartiles had a higher prevalence of education level completed high school, current smoker, former drinker, hypertension and CVD (*p* < 0.001).

**Table 2:** Characteristics of the participants according to the quartiles of SII.

Variable	Quartiles of SII				χ2/t	*p* value
	Q1	Q2	Q3	Q4		
Age	43.96 ± 16.29	44.78 ± 16.08	46.35 ± 16.02	47.24 ± 16.16	4.15	<0.001
Gender					18.90	<0.001
Female	953 (0.41)	1,007 (0.45)	1,154 (0.49)	1,238 (0.55)		
Male	1,378 (0.59)	1,313 (0.55)	1,171 (0.51)	1,087 (0.45)		
Race					20.76	<0.001
Non-Hispanic Black	680 (0.16)	392 (0.09)	370 (0.08)	320 (0.07)		
Non-Hispanic White	864 (0.63)	1,089 (0.72)	1,142 (0.73)	1,272 (0.76)		
Mexican American	314 (0.07)	376 (0.08)	354 (0.07)	317 (0.06)		
Other Hispanic and other	473 (0.13)	463 (0.11)	459 (0.12)	416 (0.11)		
Education level					4.87	<0.001
Less than high school	436 (0.13)	450 (0.12)	458 (0.13)	458 (0.14)		
Completed high school	509 (0.21)	490 (0.20)	516 (0.22)	590 (0.26)		
More than high school	1,386 (0.67)	1,380 (0.68)	1,351 (0.65)	1,277 (0.60)		
Marital status					5.43	<0.001
Never married	516 (0.23)	419 (0.18)	438 (0.18)	403 (0.17)		
Partner or married	1,432 (0.64)	1,503 (0.68)	1,427 (0.65)	1,384 (0.63)		
Separated or divorced	281 (0.11)	278 (0.10)	333 (0.12)	367 (0.15)		
Widowed	102 (0.03)	120 (0.04)	127 (0.04)	171 (0.05)		
PIR					2.83	0.013
< 1	461 (0.13)	405 (0.11)	412 (0.11)	450 (0.13)		
1–4	1,215 (0.49)	1,178 (0.46)	1,196 (0.48)	1,264 (0.50)		
> 4	655 (0.38)	737 (0.43)	717 (0.41)	611 (0.37)		
PA					7.60	<0.001
< 600	479 (0.18)	491 (0.21)	588 (0.24)	614 (0.27)		
600–3,000	915 (0.40)	933 (0.41)	920 (0.41)	901 (0.40)		
> 3,000	937 (0.42)	896 (0.38)	817 (0.36)	810 (0.32)		
Smoking status					12.54	<0.001
Never	1,367 (0.59)	1,338 (0.58)	1,276 (0.56)	1,114 (0.47)		
Former smoker	563 (0.25)	576 (0.25)	573 (0.25)	612 (0.26)		
Current smoker	401 (0.16)	406 (0.16)	476 (0.19)	599 (0.27)		
Alcohol use					5.99	<0.001
Never	282 (0.10)	271 (0.10)	251 (0.08)	246 (0.08)		
Former drinker	314 (0.10)	300 (0.10)	330 (0.11)	391 (0.15)		
Current drinker	1,735 (0.80)	1,749 (0.80)	1,744 (0.81)	1,688 (0.77)		
Hypertension					9.58	<0.001
No	1,472 (0.69)	1,504 (0.69)	1,416 (0.65)	1,307 (0.60)		
Yes	859 (0.31)	816 (0.31)	909 (0.35)	1,018 (0.40)		
Hyperlipidemia					14.04	<0.001
No	815 (0.38)	690 (0.33)	640 (0.27)	616 (0.27)		
Yes	1,516 (0.62)	1,630 (0.67)	1,685 (0.73)	1,709 (0.73)		
CVD					8.94	<0.001
No	2,144 (0.94)	2,153 (0.94)	2,138 (0.93)	2,046 (0.91)		
Yes	187 (0.06)	167 (0.06)	187 (0.07)	279 (0.09)		
DM					13.60	<0.001
No	1,980 (0.90)	1,934 (0.89)	1,927 (0.87)	1,841 (0.93)		
Yes	351 (0.10)	386 (0.11)	398 (0.13)	484 (0.17)		
HEI	51.80 ± 13.96	51.09 ± 13.90	50.71 ± 14.00	48.81 ± 13.00	−4.11	<0.001
eGFR	97.95 ± 19.84	96.80 ± 20.06	96.02 ± 20.28	94.83 ± 21.30	−3.13	0.002
TyG	8.45 ± 0.69	8.55 ± 0.65	8.61 ± 0.65	8.62 ± 0.65	5.70	<0.001
HbA1c	5.49 ± 0.84	5.52 ± 0.83	5.55 ± 0.88	5.61 ± 0.90	2.42	0.017
BMI	27.43 ± 5.73	28.06 ± 6.03	28.99 ± 6.48	29.89 ± 7.51	8.64	<0.001
WC	95.04 ± 15.21	97.04 ± 15.33	99.31 ± 15.98	101.44 ± 17.77	9.15	<0.001
LAP	48.02 ± 63.00	51.91 ± 49.45	57.44 ± 53.51	61.03 ± 56.90	3.47	0.001
VAI	1.75 ± 2.53	1.83 ± 1.93	2.00 ± 2.09	2.06 ± 2.21	2.12	0.037
Ln-SII	5.45 ± 0.31	5.93 ± 0.09	6.25 ± 0.10	6.75 ± 0.28	12.10	<0.001

Percentage and mean ± standard deviation were weighted. SII, systemic immune-inflammation index; Q1, first quartile; Q2, second quartile; Q3, third quartile; Q4, fourth quartile; PIR, poverty index ratio; PA, physical activity; CVD, cardiovascular disease; HEI, healthy eating index; eGFR, estimated glomerular filtration rate; TyG, triglyceride and glucose index; HbA1c, glycohemoglobin; BMI, body mass index; WC, waist circumference; LAP, lipid accumulation product index; VAI, visceral adiposity index; DM, diabetes mellitus; Ln, natural logarithm.

### 3.3 Association between SII and DM

**Figure 2:**
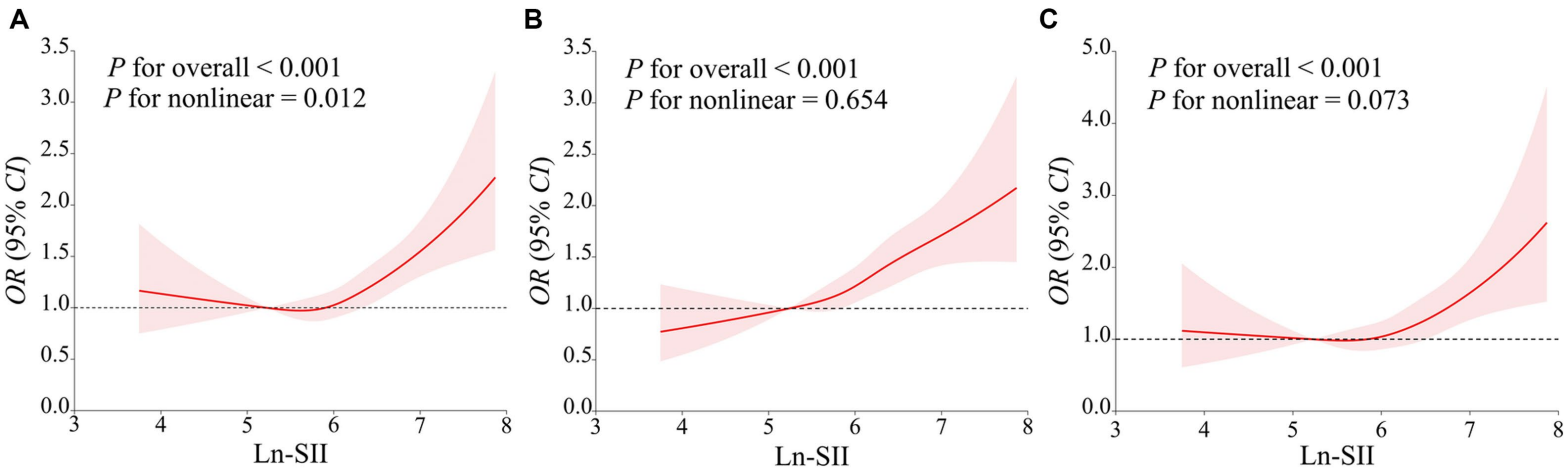
The restricted cubic spline curve was used to model the relationship between Ln-SII and the risk of DM **(A–C)**. **(A)** did not adjust any covariates; **(B)** adjusted for age, gender, race, education level, marital status, PIR; **(C)** further adjusted for age, gender, race, education level, marital status, PIR, PA, smoking status, alcohol use, hypertension, hyperlipidemia, CVD, HEI, eGFR, TyG and HbA1c.

In all three models, SII demonstrated a positive correlation with the risk of DM in the third quartile [OR: 1.39 (95% CI: 1.04, 1.78)] and the fourth quartile [OR: 1.73 (95% CI: 1.28, 2.33)] compared to the first quartile. In Model 2, a positive association between SII and DM risk was also observed in the second quartile [OR: 1.30 (95% CI: 1.01, 1.69)]. Additionally, a significant dose–response relationship was found across all three models (*p*-trend <0.05). The analysis with SII to increase by 1-SD and the Ln-SII yielded similar results. As depicted in Figure 2, the restricted cubic spline curve confirms a non-linear association between Ln-SII and the risk of DM in Model 1 (*p-*nonlinear <0.05). However, a significant linear dose–response relationship is observed between Ln-SII and the risk of DM in Model 2 (*p-*nonlinear = 0.654) and Model 3 (*p-*nonlinear = 0.073) (see Table 3).

**Table 3:** Risk of DM according to quartiles of SII.

Variable	Model 1^a^		Model 2^b^		Model 3^c^	
	OR (95% CI)	*p* value	OR (95% CI)	*p* value	OR (95% CI)	*p* value
Quartiles of SII						
Q1	Ref. (1.00)		Ref. (1.00)		Ref. (1.00)	
Q2	1.21 (0.94, 1.54)	0.132	1.30 (1.01, 1.69)	0.046	1.22 (0.93, 1.61)	0.146
Q3	1.42 (1.16, 1.74)	<0.001	1.45 (1.16, 1.81)	0.001	1.36 (1.04, 1.78)	0.026
Q4	1.86 (1.54, 2.26)	<0.001	1.88 (1.51, 2.33)	<0.001	1.73 (1.28, 2.33)	<0.001
*P*-trend	<0.001		<0.001		0.001	
Per 1-SD	1.23 (1.15, 1.31)	<0.001	1.20 (1.11, 1.29)	<0.001	1.25 (1.11, 1.40)	<0.001
Ln-SII	1.58 (1.37, 1.81)	<0.001	1.54 (1.32, 1.79)	<0.001	1.52 (1.20, 1.93)	0.001

All estimates were weighted. Logistic regression models were used to estimate OR and 95% CIs, and trend test based on within-group medians. SII, systemic immune-inflammation index; DM, diabetes mellitus; OR, odds ratio; CI, confidence interval; Q1, first quartile; Q2, second quartile; Q3, third quartile; Q4, fourth quartile; SD, standard deviation; Ln, natural logarithm.

a Model 1: Univariate analysis.

b Model 2: adjusted for age, gender, race, education level, marital status, PIR.

c Model 3: adjusted for age, gender, race, education level, marital status, PIR, PA, smoking status, alcohol use, hypertension, hyperlipidemia, CVD, HEI, eGFR, TyG and HbA1c.

### 3.4 Correlation of obesity indicators and their association with SII and DM

**Figure 3:**
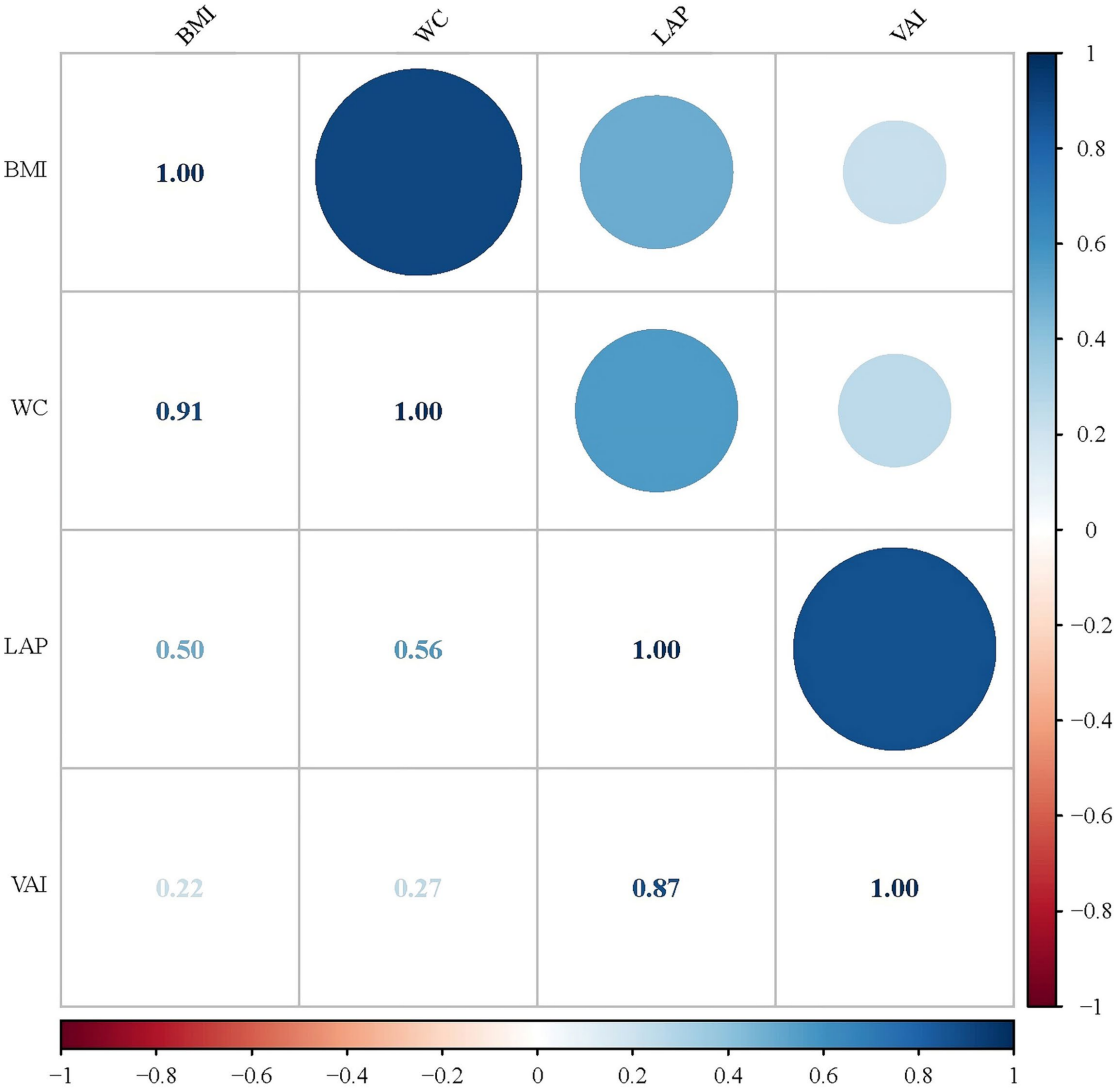
Correlations of the four obesity indicators.

As shown in Figure 3, we investigated the correlations of the four obesity indicators. The most pronounced correlation was observed between BMI and WC (*r*-value = 0.91). Additionally, a notable association was also identified between LAP and VAI (*r*-value = 0.87). The results of linear regression analyses between obesity indicators and SII are presented in Table 4. In Models 1 and 2, all obesity indicators (BMI, WC, LAP and VAI) were correlated with SII (*p*-trend <0.05). In Model 3, only BMI and WC were associated with SII (*p*-trend <0.001). Similar results were obtained for Ln-SII analysis, but in Model 3, three obesity indicators (BMI, WC, and VAI) were correlated with Ln-SII (*p*-trend <0.05). Table 5 presents the logistic regression analysis between different obesity indicators and DM. We found that in Models 1 and 2, participants with DM exhibit a higher tendency toward obesity in all obesity indicators (BMI, WC, LAP and VAI) (*p* < 0.001). However, in model 3, only BMI and WC were significantly associated with the risk of DM (*p* < 0.001).

**Table 4:** Associations between obesity indicators and SII.

Variable		Quartiles of SII				*p*-trend	Ln-SII	
		Q1	Q2 (95% *CI*)	Q3 (95% *CI*)	Q4 (95% *CI*)		*β* (95% *CI*)	*p*
Model 1^a^	BMI	Ref. (0.00)	0.64 (0.24, 1.03)	1.56 (1.12, 2.01)	2.46 (1.91, 3.00)	<0.001	1.73 (1.33, 2.13)	<0.001
	WC	Ref. (0.00)	2.00 (0.95, 3.06)	4.27 (3.06, 5.48)	6.40 (5.10, 7.69)	<0.001	4.50 (3.53, 5.46)	<0.001
	LAP	Ref. (0.00)	3.89 (−0.19, 7.98)	9.43 (4.85, 14.00)	13.02 (8.30, 17.73)	<0.001	9.30 (6.11, 12.50)	<0.001
	VAI	Ref. (0.00)	0.07 (−0.09, 0.24)	0.25 (0.07, 0.42)	0.31 (0.14, 0.48)	<0.001	0.22 (0.11, 0.32)	<0.001
Model 2^b^	BMI	Ref. (0.00)	0.80 (0.41, 1.19)	1.71 (1.28, 2.15)	2.60 (2.07, 3.13)	<0.001	1.86 (1.47, 2.25)	<0.001
	WC	Ref. (0.00)	2.26 (1.24, 3.28)	4.52 (3.40, 5.64)	6.79 (5.53, 8.04)	<0.001	4.79 (3.86, 5.73)	<0.001
	LAP	Ref. (0.00)	3.12 (−0.95, 7.19)	8.36 (3.78, 12.93)	11.75 (7.00, 16.49)	<0.001	8.27 (5.05, 11.50)	<0.001
	VAI	Ref. (0.00)	0.03 (−0.13, 0.20)	0.19 (0.01, 0.37)	0.23 (0.06, 0.41)	0.002	0.16 (0.05, 0.27)	0.004
Model 3^c^	BMI	Ref. (0.00)	0.50 (0.15, 0.85)	1.19 (0.82, 1.56)	1.97 (1.47, 2.47)	<0.001	1.40 (1.05, 1.75)	<0.001
	WC	Ref. (0.00)	1.45 (0.57, 2.34)	3.10 (2.13, 4.06)	4.99 (3.83, 6.15)	<0.001	3.49 (2.67, 4.31)	<0.001
	LAP	Ref. (0.00)	−2.57 (−5.04, −0.10)	−1.47 (−4.09, 1.14)	1.37 (−1.57, 4.31)	0.080	0.86 (−1.06, 2.79)	0.376
	VAI	Ref. (0.00)	−0.18 (−0.29, −0.07)	−0.17 (−0.29, −0.06)	−0.14 (−0.26, −0.03)	0.069	−0.11 (−0.18, −0.03)	0.005

All estimates were weighted. Linear regression models were used to estimate β and 95% CIs, and trend test based on within-group medians. SII, systemic immune-inflammation index; Ln, natural logarithm; T1, first tertile; T2, second tertile; T3, third tertile; CI, confidence interval; BMI, body mass index; WC, waist circumference; LAP, lipid accumulation product index; VAI, visceral adiposity index; Ln, natural logarithm.

a Model 1: univariate analysis.

b Model 2: adjusted for age, gender, race, education level, marital status, PIR.

c Model 3: adjusted for age, gender, race, education level, marital status, PIR, PA, smoking status, alcohol use, hypertension, hyperlipidemia, CVD, HEI, eGFR, TyG and HbA1c.

**Table 5:** Associations between obesity indicators and DM.

Variable	Model 1^a^		Model 2^b^		Model 3^c^	
	OR (95% CI)	*p* value	OR (95% CI)	*p* value	OR (95% CI)	*p* value
BMI	1.10 (1.08, 1.11)	<0.001	1.12 (1.10, 1.13)	<0.001	1.04 (1.02, 1.06)	<0.001
WC	1.05 (1.05, 1.05)	<0.001	1.05 (1.05, 1.06)	<0.001	1.02 (1.01, 1.03)	<0.001
LAP	1.01 (1.01, 1.01)	<0.001	1.01 (1.01, 1.01)	<0.001	1.00 (1.00, 1.00)	0.345
VAI	1.21 (1.16, 1.25)	<0.001	1.23 (1.18, 1.28)	<0.001	0.95 (0.86, 1.06)	0.352

All estimates were weighted. Logistic regression models were used to estimate OR and 95% CIs. DM, diabetes mellitus; OR, odds ratio; CI, confidence interval; BMI, body mass index; WC, waist circumference; LAP, lipid accumulation product index; VAI, visceral adiposity index.

a Model 1: univariate analysis.

b Model 2: adjusted for age, gender, race, education level, marital status, PIR.

c Model 3: adjusted for age, gender, race, education level, marital status, PIR, PA, smoking status, alcohol use, hypertension, hyperlipidemia, CVD, HEI, eGFR, TyG and HbA1c.

### 3.5 Mediating role of obesity indicators

**Figure 4:**
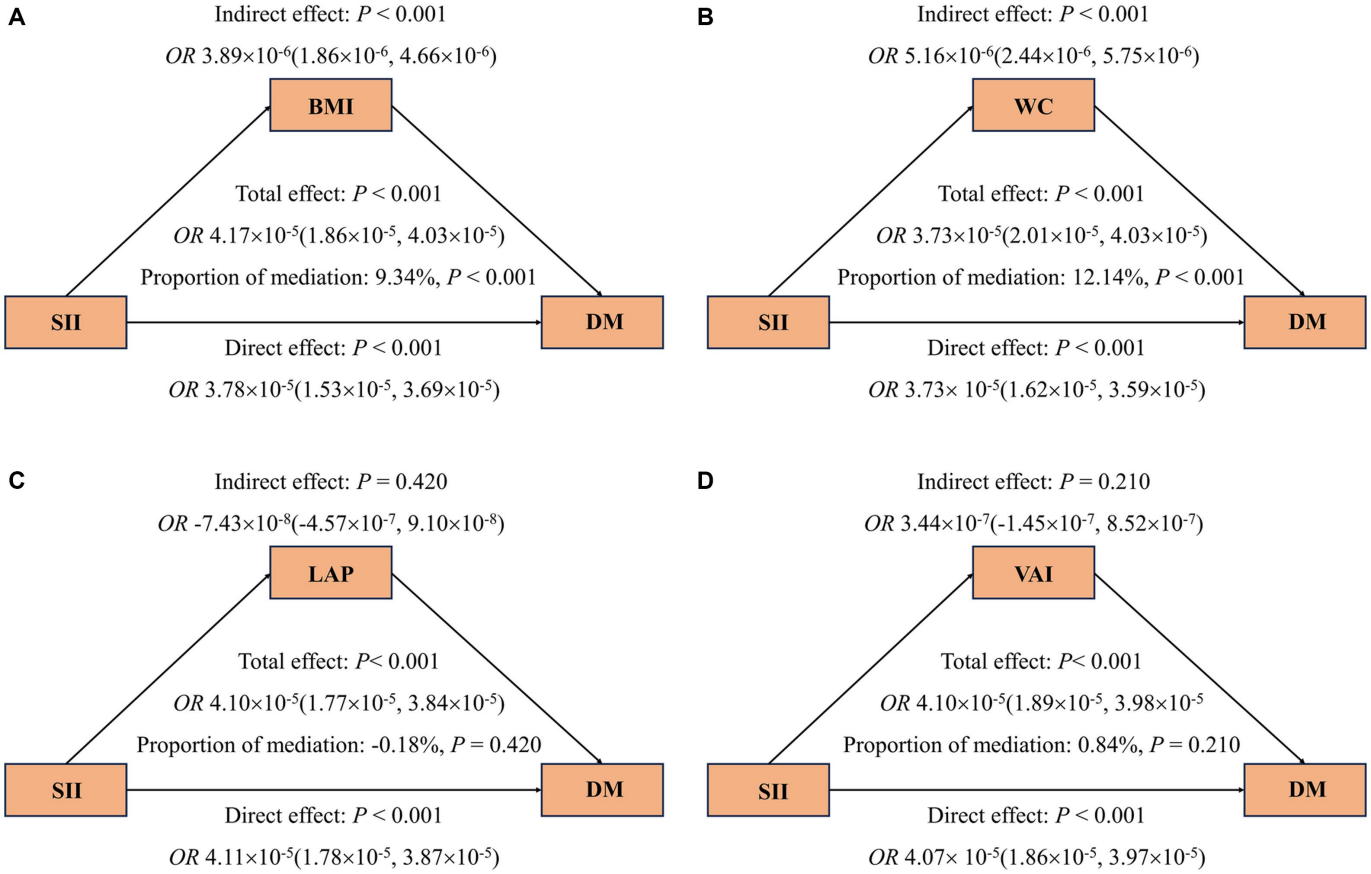
Mediating effects of obesity indicators on the association between SII and odds ratios of DM **(A–D)**. The 95% CI of these estimates was computed using the bootstrap method (1,000 samples). In all mediation analyses, adjustments were made for the following covariates: age, gender, race, education level, marital status, PIR, PA, smoking status, alcohol use, hypertension, hyperlipidemia, CVD, HEI, eGFR, TyG, and HbA1c.

**Figure 5:**
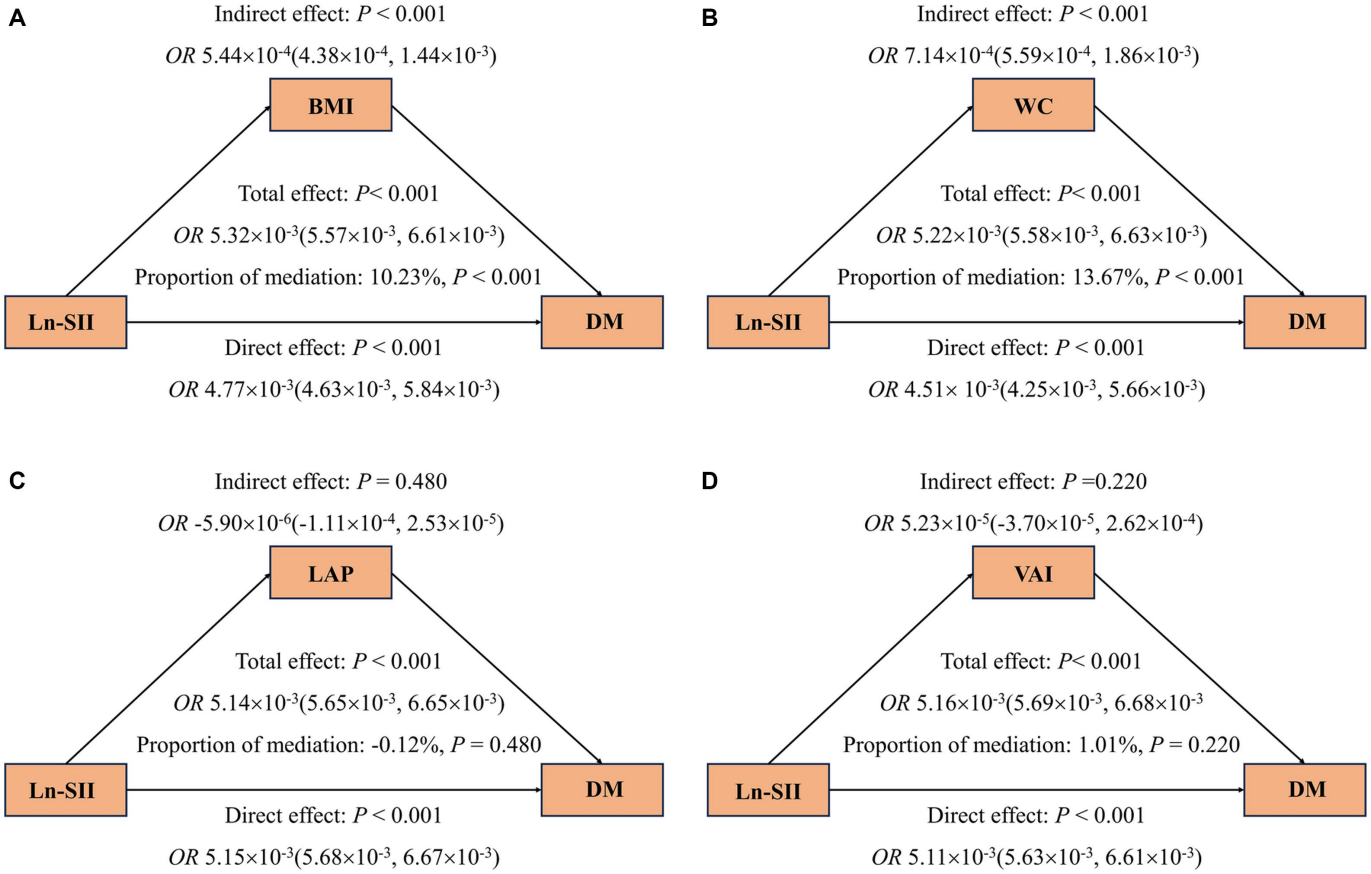
Mediating effects of obesity indicators on the association between Ln-SII and odds ratios of DM **(A–D)**. The 95% CI of these estimates was computed using the bootstrap method (1,000 samples). In all mediation analyses, adjustments were made for the following covariates: age, gender, race, education level, marital status, PIR, PA, smoking status, alcohol use, hypertension, hyperlipidemia, CVD, HEI, eGFR, TyG and HbA1c.

The mediation analysis results depicted in Figure 4 show that, an increase in SII is consistently associated with a higher risk of DM (A-D), even after adjusting for covariates. Furthermore, the findings suggest that part of the association between SII and DM is mediated by BMI and WC, with respective mediation proportions of 9.34% and 12.14% (*p* < 0.001). Likewise, Figure 5 reveals that when analyzing the data using Ln-SII, BMI and WC also act as mediators in the relationship between Ln-SII and DM, with mediation proportions of 10.23% and 13.67%, respectively (*p* < 0.001). However, whether it is SII or Ln-SII, there is no mediating effect between LAP and VAI.

### 3.6 Sensitivity analyses

After redividing the intervals of the SII into tertiles, all sensitivity analyses yielded similar results (Supplementary Tables S1–S3). The positive correlation between SII and the risk of DM did not exhibit significant changes. In models 1 and 2, compared to the first tertile, SII showed a positive association with the risk of DM in both the second tertile [OR: 1.32 (95% CI: 1.01–1.64)] and the third tertile [OR: 1.73 (95% CI: 1.43–2.09)]. Simultaneously, in model 3, SII remained positively correlated with the risk of DM in the third tertile [OR: 1.64 (95% CI: 1.27–2.11)] (Supplementary Table S2). Furthermore, the results of the linear regression analysis between obesity indicators and SII indicated that, in model 3, three indices (BMI, WC, and VAI) were associated with SII (*p*-trend <0.05) (Supplementary Table S3).

## 4 Discussion

This study finally included 9,301 participants from the NHANES 2005–2018 cohort for analysis, including 4,352 females and 4,949 males. Of these, 1,619 patients had DM. We found that SII was an independent risk factor for DM, and there was a positive relationship between the Ln-SII and the risk of DM, with a non-linear association in models 1, and a linear association in model 2 and 3. Furthermore, obesity indicators (BMI, WC, LAP and VAI) were higher in DM patients, and these indicators gradually increased as the SII increased. Among them, BMI and WC had a mediating effect on the relationship between SII and DM.

As a novel inflammatory marker, SII is a valuable tool in medical research and clinical practice, as it assesses the balance between systemic inflammation and immune response in an individual, offering insights into an individual’s inflammatory status and immune system activity (22, 23). To the best of our knowledge, our study is the first to report that the SII level of patients with DM was higher than that of healthy controls by using the NHANES database. However, several research studies have demonstrated that SII is associated with various DM-related conditions. Wang et al. reported that SII levels were significantly higher in T2DM patients who had diabetic kidney disease (DKD) compared to those without DKD, and SII levels were associated with an increased likelihood of DKD in T2DM patients (24). In patients with diabetic macular edema (DME), SII levels were significantly higher than those non-DME patients, and SII was considered a potential biomarker for diagnosing DME (25). Another study demonstrated that SII was significantly elevated in diabetic foot infection patients with osteomyelitis compared to those with cellulitis, with the area under the curve (AUC) value of 0.687, indicating the potential of SII as an effective and novel inflammatory marker for predicting diabetic foot osteomyelitis (26). Moreover, SII levels were associated with an increased risk of depression in patients with DM (27). These findings highlight the critical research significance of SII in DM and its associated conditions. As we all know, Inflammation can manifest as both a cause and a consequence of DM, creating a complex interplay between the two. On one hand, chronic inflammation in the body may contribute to the development of T2DM by affecting insulin sensitivity and disrupting normal metabolic processes (16). Conversely, hyperglycemia, a hallmark of DM, can activate immune responses and trigger inflammatory pathways (28). In our study, SII demonstrated a positive correlation with the risk of DM and a significant dose–response relationship was found, which persisted after adjusting for confounding factors. Similar results were observed for Ln-SII, indicating that SII is an independent risk factor for DM. Therefore, it is possible to prevent DM by managing systemic inflammation, which corresponds to the current understanding of the relationship between inflammation and DM (29).

In addition, we found that obesity indicators (BMI, WC, LAP, and VAI) were all also higher in patients with DM than healthy controls, which was consistent with previous research (30, 31). Simultaneously, we also observed a robust interrelation among these four obesity indicators in DM patients. It is well-known that obesity is an important risk factor for the development of DM (32). Looker et al. (33) observed that before the diagnosis of T2DM, individuals experienced consistent weight increases, with the mean BMI rising by 0.43 to 0.71 kg/m^2^ per year. As for WC, a two-sample Mendelian randomization study had reported that the higher WC increased the risk of T2DM among the European population (34). According to our study findings, in both Models 1 and 2, all obesity indicators (BMI, WC, LAP, and VAI) were correlated with SII, and the increase in each of these four obesity indicators elevated the risk of developing DM. However, in Model 3, only BMI and WC were correlated with SII and significantly associated with the risk of DM. This suggests that the association between LAP and VAI with SII and DM may be influenced by confounding factors, including PA, smoking status, alcohol use, hypertension, hyperlipidemia, CVD, HEI, eGFR, TyG and HbA1c.

The VAI was originally introduced by Amato et al. (35) as an indicator to assess cardiometabolic risk in a healthy population, revealing a significant inverse correlation with insulin sensitivity. A cohort study conducted on a Chinese population revealed that higher VAI was independently associated with DM risk and had a higher overall DM diagnostic ability than BMI and WC in Chinese adults, and this conclusion remains consistent after accounting for various confounding factors (30). Similar findings had also been observed in Qatari population (36). LAP, devised for the U.S. National Health and Nutrition Examination Survey, has been employed as an indicator of central obesity (37). Additionally, it is suggested as a marker associated with insulin resistance and impaired glucose tolerance (38). Based on the results of a national physical examination project in Urumqi, China, Tian et al. (39) discovered that the subjects with higher LAP levels had a higher risk of DM, and LAP performed better than BMI when used as a tool for DM diagnosis. These are somewhat different from our results, and we speculate that the difference may be attributed to the different ethnicity, lifestyles, dietary patterns and living area of the study populations. In summary, obesity is also a risk factor for diabetes, and effective management of obesity can prevent the occurrence of DM (10).

It is widely recognized that inflammation and obesity exhibit intricate interactions (40). Adipose tissue can secrete pro-inflammatory molecules, creating a chronic low-grade inflammatory state in obesity, while inflammation can contribute to the dysregulation of appetite and metabolism, leading to increased food intake and thereby accelerating obesity (16–18). As an inflammatory marker, we have found that SII was also associated with obesity indicators. In addition to our research findings, Thavaraputta et al. (41) revealed that several inflammatory markers, including SII, were significantly and positively correlated with BMI in healthy adult participants from NHANES 2011–2016. Similar findings have been observed by Duman et al. (42), where SII was significantly positively correlated with BMI in patients with diabetic kidney injury. Therefore, the SII and obesity indicators may have a mutually reinforcing relationship. To further investigate whether the relationship between SII and DM can be mediated by obesity, we conducted a mediation analysis. Our results showed that an increase in SII was consistently associated with a higher risk of DM, and the link between SII and DM was mediated by BMI and WC, with respective mediation proportions of 9.34% and 12.14%. Moreover, BMI and WC also acted as mediators in the relationship between Ln-SII and DM, with mediation proportions of 10.23% and 13.67%, respectively. This suggests that obesity may play a critical mediating role in the relationship between inflammation and DM, which confirms that alleviating systemic inflammation and weight loss may be useful in preventing DM.

Overall, our study results suggest a potential intricate interplay between SII and obesity indicators (BMI, WC, LAP and VAI). Increased SII and elevated obesity indicators were associated with an increased risk of DM, with BMI and WC acting as mediators in the relationship between SII and DM.

The present study has several strengths. Firstly, we explore the association between SII and DM based on the large sample data with national representation. Secondly, we used a variety of obesity indicators (BMI, WC, VAI, and LAP) to investigate the potential mediating role of obesity in the relationship between SII and DM. The utilization of diverse obesity indicators allows for a multifaceted assessment of obesity from various angles. In particular, VAI and LAP encompass metabolism-related parameters, which makes our analysis more comprehensive. Finally, we adjusted for many key confounding variables in our study and conducted sensitivity analysis to validate our conclusions.

However, there are some limitations in our research. First, due to the nature of observational study design, we cannot establish a definitive causal relationship. Second, the findings of this current study were derived from American adults, and thus, the characteristics of other populations may not be comprehensively represented. Third, although we adjusted for many potential confounders, we cannot entirely eliminate the possibility of unmeasured confounders.

## 5 Conclusion

In summary, this study investigated the relationship among SII, various obesity indicators, and DM. The findings demonstrated that elevated SII levels were independently associated with the risk of DM, with BMI and WC playing substantial roles in mediating the relationship between SII and the risk of DM, suggesting proper control of obesity can be effective to reduce the effects of inflammation on DM.

## Data availability statement

Publicly available datasets were analyzed in this study. This data can be found at: https://www.cdc.gov/nchs/nhanes/index.htm.

## Ethics statement

The NHANES protocol is approved by the National Center for Health Statistics Institutional Review Board, and written informed consent has been obtained. Written informed consent for participation was not required for this study in accordance with the national legislation and the institutional requirements.

## Author contributions

YC: Conceptualization, Data curation, Methodology, Visualization, Writing – original draft. RH: Conceptualization, Data curation, Methodology, Writing – original draft. ZM: Methodology, Writing – original draft. HC: Data curation, Writing – original draft. JZ: Visualization, Writing – original draft. LZ: Writing – original draft. ZY: Writing – original draft. HY: Investigation, Writing – review & editing. DK: Supervision, Writing – review & editing. YD: Funding acquisition, Investigation, Resources, Supervision, Writing – review & editing.
