# Supplementary material for: Association between systemic immune-inflammatory index and diabetes mellitus: mediation analysis involving obesity indicators in the NHANES
Source: Front Public Health. 2024 Jan 10;11:1331159. doi: 10.3389/fpubh.2023.1331159 (PMC10806151; doi:10.3389/fpubh.2023.1331159)
Supplement: Supplementary file 1 [file Table_1.DOCX]

Supplementary Material

# Supplementary Tables

**Table S1** **Characteristics of the participants according to the tertiles of SII.**

| Variable | Tertiles of SII | | | *χ^2^/t* | *P* value |
| --- | --- | --- | --- | --- | --- |
|  | T1 | T2 | T3 |  |  |
| Age | 44.22 ± 16.39 | 45.32 ± 15.79 | 47.21 ± 16.25 | 4.32 | <0.001 |
| Gender |  |  |  | 30.61 | <0.001 |
| Female | 1,268 (0.41) | 1,448 (0.48) | 1,636 (0.54) |  |  |
| Male | 1,834 (0.59) | 1,651 (0.52) | 1,464 (0.46) |  |  |
| Race |  |  |  | 27.22 | <0.001 |
| Non-Hispanic Black | 829 (0.15) | 500 (0.08) | 433 (0.07) |  |  |
| Non-Hispanic White | 1,207 (0.65) | 1,482 (0.73) | 1,678 (0.76) |  |  |
| Mexican American | 442 (0.08) | 502 (0.08) | 417 (0.06) |  |  |
| Other Hispanic and other | 624 (0.13) | 615 (0.11) | 572 (0.11) |  |  |
| Education level |  |  |  | 6.49 | <0.001 |
| Less than high school | 590 (0.12) | 611 (0.12) | 601 (0.14) |  |  |
| Completed high school | 665 (0.20) | 664 (0.21) | 776 (0.26) |  |  |
| More than high school | 1,847 (0.67) | 1,824 (0.67) | 1,723 (0.61) |  |  |
| Marital status |  |  |  | 6.47 | <0.001 |
| Never married | 665 (0.22) | 562 (0.18) | 549 (0.17) |  |  |
| Partner or married | 1,925 (0.65) | 1,962 (0.67) | 1,859 (0.64) |  |  |
| Separated or divorced | 372 (0.10) | 411 (0.11) | 476 (0.14) |  |  |
| Widowed | 140 (0.03) | 164 (0.04) | 216 (0.05) |  |  |
| PIR |  |  |  | 3.83 | 0.006 |
| < 1 | 608 (0.13) | 521 (0.10) | 599 (0.13) |  |  |
| 1-4 | 1,601 (0.48) | 1,601 (0.48) | 1,651 (0.49) |  |  |
| > 4 | 893 (0.39) | 977 (0.42) | 850 (0.38) |  |  |
| PA |  |  |  | 9.28 | <0.001 |
| < 600 | 630 (0.19) | 751 (0.22) | 791 (0.26) |  |  |
| 600-3000 | 1,213 (0.40) | 1,232 (0.41) | 1,224 (0.41) |  |  |
| > 3000 | 1,259(0.41) | 1,116 (0.37) | 1,085 (0.33) |  |  |
| Smoking status |  |  |  | 18.50 | <0.001 |
| Never | 1,796 (0.58) | 1,767 (0.57) | 1,532 (0.49) |  |  |
| Former smoker | 769 (0.26) | 754 (0.25) | 801 (0.26) |  |  |
| Current smoker | 537 (0.16) | 578 (0.18) | 767 (0.25) |  |  |
| Alcohol use |  |  |  | 8.11 | <0.001 |
| Never | 367 (0.10) | 352 (0.09) | 331 (0.08) |  |  |
| Former drinker | 418 (0.10) | 416 (0.10) | 501 (0.14) |  |  |
| Current drinker | 2,317 (0.80) | 2,331 (0.81) | 2,268 (0.77) |  |  |
| Hypertension |  |  |  | 12.63 | <0.001 |
| No | 1,964 (0.69) | 1,960 (0.67) | 1,775 (0.61) |  |  |
| Yes | 1,138 (0.31) | 1,139 (0.33) | 1,325 (0.39) |  |  |
| Hyperlipidemia |  |  |  | 22.57 | <0.001 |
| No | 1,060 (0.37) | 900 (0.30) | 801 (0.26) |  |  |
| Yes | 2,042 (0.63) | 2,199 (0.70) | 2,299 (0.74) |  |  |
| CVD |  |  |  | 17.02 | <0.001 |
| No | 2,846 (0.90) | 2,882 (0.88) | 2,753 (0.84) |  |  |
| Yes | 256 (0.10) | 217 (0.12) | 347 (0.16) |  |  |
| DM |  |  |  | 18.76 | <0.001 |
| No | 2623 (0.90) | 2576 (0.88) | 2483 (0.84) |  |  |
| Yes | 479 (0.10) | 523 (0.12) | 617 (0.16) |  |  |
| HEI | 51.74 ± 13.91 | 50.83 ± 13.90 | 49.25 ± 13.37 | -4.71 | <0.001 |
| eGFR | 97.63 ± 20.22 | 96.68 ± 19.89 | 94.88 ± 21.02 | -2.90 | 0.005 |
| TyG | 8.47 ± 0.68 | 8.58 ± 0.65 | 8.62 ± 0.64 | 6.41 | <0.001 |
| HbA1c | 5.50 ± 0.86 | 5.53 ± 0.86 | 5.60 ± 0.88 | 2.76 | 0.007 |
| BMI | 27.55 ± 5.72 | 28.52 ± 6.39 | 29.70 ± 7.24 | 8.84 | <0.001 |
| WC | 95.55 ± 15.23 | 98.10 ± 15.75 | 100.98 ± 17.34 | 8.88 | <0.001 |
| LAP | 49.11 ± 60.84 | 54.71 ± 52.57 | 59.99 ± 54.23 | 3.82 | <0.001 |
| VAI | 1.78 ± 2.46 | 1.91 ± 2.05 | 2.04 ± 2.08 | 2.38 | 0.019 |
| Ln-SII | 5.54 ± 0.31 | 6.08 ± 0.12 | 6.65 ± 0.29 | 13.52 | <0.001 |

Rate and mean ± standard deviation were weighted.

Abbreviations: SII, systemic immune-inflammation index; T1, first tertile; T2, second tertile; T3, third tertile; PIR, poverty index ratio; PA, physical activity; CVD, cardiovascular disease; HEI, healthy eating index; eGFR, estimated glomerular filtration rate; TyG, triglyceride and glucose index; HbA1c, glycohemoglobin; BMI, body mass index; WC, waist circumference; LAP, lipid accumulation product index; VAI, visceral adiposity index; DM, diabetes mellitus; Ln, natural logarithm.

**Table S2** **Risk of DM according to tertiles of SII.**

| Variable | Model 1 ^a^ | | Model 2 ^b^ | | Model 3 ^c^ | |
| --- | --- | --- | --- | --- | --- | --- |
|  | *OR* (95% *CI*) | *P* value | *OR* (95% *CI*) | *P* value | *OR* (95% *CI*) | *P* value |
| Tertiles of SII |  |  |  |  |  |  |
| T1 | Ref. (1.00) |  | Ref. (1.00) |  | Ref. (1.00) |  |
| T2 | 1.24 (1.02, 1.50) | 0.033 | 1.32 (1.01, 1.64) | 0.011 | 1.15 (0.88, 1.49) | 0.308 |
| T3 | 1.73 (1.46, 2.05) | <0.001 | 1.73 (1.43, 2.09) | <0.001 | 1.64 (1.27, 2.11) | <0.001 |
| *P*-trend | <0.001 |  | <0.001 |  | <0.001 |  |

All estimates were weighted. Logistic regression models were used to estimate *OR* and 95% *CIs,* and trend test based on within-group medians.

Abbreviations: SII, systemic immune-inflammation index; DM, diabetes mellitus; *OR*, odds ratio; *CI*, confidence interval; T1, first tertile; T2, second tertile; T3, third tertile; SD, standard deviation; Ln, natural logarithm.

^a^ Molde 1: Univariate analysis;

^b^ Molde 2: Adjusted for age, gender, race, education level, marital status, PIR.

^c^ Molde 3: Adjusted for age, gender, race, education level, marital status, PIR, PA, smoking status, alcohol use, hypertension, hyperlipidemia, CVD, HEI, eGFR, TyG and HbA1c.

**Table S3 Associations between anthropometric and biochemical indices and SII**

| Variable | | Quartiles of SII | | | | | *P*-trend |
| --- | --- | --- | --- | --- | --- | --- | --- |
|  |  | T1 | T2 (95% *CI*) | *P* value | T3 (95% *CI*) | *P* value |  |
| Model 1^a^ | BMI | Ref. (0.00) | 0.98 (0.57, 1.39) | <0.001 | 2.16 (1.69, 2.62) | <0.001 | <0.001 |
|  | WC | Ref. (0.00) | 2.55 (1.53, 3.58) | <0.001 | 5.43 (4.28, 6.58) | <0.001 | <0.001 |
|  | LAP | Ref. (0.00) | 5.60 (1.99, 9.21) | 0.003 | 10.88 (7.04, 14.72) | <0.001 | <0.001 |
|  | VAI | Ref. (0.00) | 0.14 (-0.01, 0.28) | 0.059 | 0.26 (0.12, 0.40) | <0.001 | <0.001 |
| Model 2^b^ | BMI | Ref. (0.00) | 1.11 (0.70, 1.52) | <0.001 | 2.28 (1.82, 2.73) | <0.001 | <0.001 |
|  | WC | Ref. (0.00) | 2.92 (1.96, 3.87) | <0.001 | 5.77 (4.65, 6.89) | <0.001 | <0.001 |
|  | LAP | Ref. (0.00) | 5.06 (1.51, 8.62) | 0.006 | 9.81 (5.92, 13.70) | <0.001 | <0.001 |
|  | VAI | Ref. (0.00) | 0.10 (-0.04, 0.25) | 0.162 | 0.20 (0.06, 0.34) | 0.007 | 0.006 |
| Model 3^c^ | BMI | Ref. (0.00) | 0.76 (0.40, 1.12) | <0.001 | 1.69 (1.28, 2.11) | <0.001 | <0.001 |
|  | WC | Ref. (0.00) | 1.95 (1.08, 2.82) | <0.001 | 4.12 (3.13, 5.12) | <0.001 | <0.001 |
|  | LAP | Ref. (0.00) | -1.60 (-3.93, 0.74) | 0.177 | 0.41 (-1.91, 2.74) | 0.724 | 0.475 |
|  | VAI | Ref. (0.00) | -0.15 (-0.24, -0.05) | 0.004 | -0.14 (-0.24, -0.04) | 0.005 | 0.009 |

All estimates were weighted. Linear regression models were used to estimate *β* and 95% *CIs*, and trend test based on within-group medians.

Abbreviations: SII, systemic immune-inflammation index; Ln, natural logarithm; T1, first tertile; T2, second tertile; T3, third tertile; *CI*, confidence interval; BMI, body mass index; WC, waist circumference; LAP, lipid accumulation product index; VAI, visceral adiposity index.

^a^ Molde 1: Univariate analysis;

^b^ Molde 2: Adjusted for age, gender, race, education level, marital status, PIR.

^c^ Molde 3: Adjusted for age, gender, race, education level, marital status, PIR, PA, smoking status, alcohol use, hypertension, hyperlipidemia, CVD, HEI, eGFR, TyG and HbA1c.
